# Public Health Threats Posed by Biofilms and Innovative Strategies for their Control

**DOI:** 10.15190/d.2024.16

**Published:** 2024-12-31

**Authors:** Syed Hamza Abbas, Shahzar Khan, Majid Shah, Jawad Aslam, Humaira Nawaz, Nadia Ilyas, Asim Gamaryani, Saba Qadir Afridi, Izaz Khan, Brekhna Shah, Kashmala Shah, Abdul Rashid, Dilawaiz Khan, Samiullah Khan

**Affiliations:** ^1^Department of Microbiology, Faculty of Biological Sciences, Quaid-i-Azam University, Islamabad, Pakistan; ^2^Saidu Medical College, Saidu Sharif, Pakistan; ^3^Saidu Group of Teaching Hospital, Saidu Sharif, Pakistan; ^4^Department of Microbiology, Kohat University of Science and Technology, Kohat, Pakistan; ^5^School of Health and society, University of Wollongong, Australia; ^6^Centre for Biotechnology and Microbiology, University of Swat, Swat, Pakistan; ^7^Khyber Medical College, Peshawar, Pakistan; ^8^Khyber Teaching Hospital, Peshawar, Pakistan; ^9^Department of Animal Sciences, Quaid -i-Azam University, Islamabad, Pakistan

**Keywords:** Biofilms, Public health threats, Bacteriophages, Biofilm disruption, Essential Oils.

## Abstract

Biofilms are communities of microorganisms that adhere to surfaces within a self-produced protective matrix. The structural complexity of biofilms and their inherent resistance to conventional antimicrobial treatments make them a significant public health challenge. These microbial communities, embedded within a self-produced extracellular matrix, are associated with numerous persistent infections, especially those occurring in healthcare settings where they colonize medical devices and chronic wounds. The effects of biofilms go beyond healthcare environments and persist in water treatment facilities, food processing plants, and nature, in which biofilms aid in pollution and transmission of disease. This review article discusses multifaceted public health complications related to biofilms and the search for existing control strategies, the process of biofilm formation, mechanisms of persistence, and limitations of traditional antimicrobial approaches. Additionally, this article explores new innovative solutions, such as bacteriophage therapy, matrix-degrading enzymes, and quorum sensing inhibitors. The potential of a combination of antimicrobial agents with biofilm-disrupting compounds for the improvement of efficacy is also paid special attention.  This review seeks to contribute to these ongoing efforts by presenting an overview of biofilm biology and assessing the efficacy of a variety of possible control strategies. Subsequently, the insights derived from this study may be used to inform future research directions and aid in the development of more effective interventions for biofilm-associated infections and contamination in various settings.

## SUMMARY

*1. Introduction*

*2. Biofilm Development*

*3. Molecular Mechanism of Biofilm Formation*

*4. Steps involved in formation of biofilms*

*5. Biofilm formation on various surfaces*

*6. Extracellular polymeric substance (EPS) formation*

*7. Regulation, Defense, and Therapeutic Challenges of Staphylococcal Biofilms*

*8. Biofilm threat to public health in developing countries*

*9. Approaches to Combat Biofilm Formation*

*10. Conclusion*

## 1. Introduction

Biofilms are communities of microorganisms that adhere to surfaces within a self-produced protective matrix. Bacteria have traditionally been studied as planktonic microorganisms. However, it is now well known that most bacteria are found in biofilms, which are composed of structured multicellular colonies encompassed by extracellular polymeric substances (EPS) ^1^. The prevalence of biofilm formation in approximately 99% of bacterial species, and the importance of biofilm research due to its adaptive advantages, including survival in nutrient-limited environments, resistance to antibiotics and disinfectants, and phenotypic variability ^2^, has led to substantial attention to the matter ^3^.

The formation of biofilms includes several successive stages: bacterial attachment to living or non-living surfaces and production of EPS, which stabilizes their three-dimensional structure. The EPS matrix is mainly composed of proteins, polysaccharides, and other large molecules of branched or linear polysaccharides, such as homopolysaccharides or heteropolysaccharides ^4^. Quorum sensing (QS) molecules enable the formation of microcolonies that eventually develop into biofilms under the influence of environmental cues (e.g. flagella, outer membrane proteins, pili, and lipopolysaccharides (LPS)). These mechanisms are crucial for understanding biotechnology and medical research because biofilms have a large impact on bacterial behavior and interaction with their environment ^5^.

It is generally associated with bacterial diseases, such as endocarditis, osteomyelitis, and with bacterial infections associated with medical devices, such as catheters and ocular implants. Biofilms have also been associated with chronic lung infections in patients with cystic fibrosis. Biofilms are difficult to destroy, and their resilience allows them to resist antimicrobial treatments at concentrations 10–1000 times the amount that kills planktonic bacteria ^6^. However, part of this resistance is due to the protective matrix, which hinders the penetration of antibiotics and aids the survival of genetically resistant cells ^7^.

Antibiotic resistance in biofilm-associated bacteria is a great public health and economic concern. Biofilms containing antibiotic-resistant bacteria are especially difficult to treat because the matrix can bind antibiotics ^8^. This issue has been exacerbated by the misuse of antibiotics, which has led to the emergence of resistant strains and novel mechanisms of resistance. Biofilms on food contact surfaces are not only a medical but also an industrial problem because food spoilage and economic losses are associated with it, and on medical implants, a serious problem of device-related infections that are costly to device/medical facilities and damage to patients ^9^.

Exploration of innovative antimicrobial strategies to counteract biofilm-associated resistance. The emergence of nanoparticles as potential materials for combating bacterial diseases is mainly because of their ability to target bacteria and effectively reduce protective biofilms. The versatility of nanomaterials in medical applications includes improving wound healing through biofilm eradication and persistent infection management ^10^. Additionally, bacterio-phages have potential because of their viral nature, which specifically infects and lyses bacteria, removes biofilms, and fights against antibiotic resistance. A range of Gram-positive and Gram-negative bacteria such as *Staphylococcus aureus*, *Escherichia coli*, *Pseudomonas aeruginosa*, and *Klebsiella pneumoniae* have been shown to be successfully inhibited by ALCs ^11^.

Another promising avenue is the essential oils. Natural antimicrobial agents include substances that disrupt the microbial cell membranes and cause cell death. Because of their ability to attack both planktonic and biofilm-associated bacteria and lack of microbial resistance, they are suitable alternatives to conventional antimicrobials ^12,13^. In addition, physical methods, including EPS matrix disruption, creation of unfavorable external conditions (such as low pH or hypoxia), and targeting stages of the biofilm life cycle, are being studied. The aim of these methods is to destroy preformed biofilms or retard their formation ^14^.

More than 65% of nosocomial infections are caused by biofilm-associated infections, which are a substantial problem for all healthcare systems worldwide, imposing an annual cost of over $1 billion in the U.S. alone. Furthermore, these infections often require removal of the infected tissues or devices, followed by additional replacement, all of which increase hospital and patient morbidity and costs. Biofilms are resilient and contribute to horizontal gene transfer, which plays a role in the development of virulent bacterial strains ^15^; hence, there is a need to develop further therapeutic strategies. However, despite their persistence, further research and increasingly imaginative approaches offer some hope of reducing the effects of biofilms on public health and the industry.

## 2. Biofilm Development

During most biofilm formation processes, individual single-celled organisms aggregate to create a community that adheres to a solid surface and is enveloped by a matrix composed of exo-polysaccharides. Microorganisms constitute less than 10% of the dry mass, while the matrix can constitute more than 90%. Various processes facilitate intimate contact, firm attachment, cell-cell interactions, and growth of diverse microbial species on a surface ^16^. A previous study has revealed that the production of microbial biofilms is influenced by both genetic and environmental variables. EPS, or extracellular polymeric substances, have earned the nickname 'the black stuff of biofilms” because of their vast array of matrix biopolymers and their challenging analysis process. EPS is mostly comprised of polysaccharides, along with other macromolecules such as proteins, lipids, and nucleic acids. Polymers such as glycopeptides, lipids, and lipopolysaccharides serve as a framework for maintaining the cohesion of biofilms ^17^.

The intricate nature of biofilm architecture and metabolic processes has resulted in the comparison of biofilms to tissues in more advanced organisms. Notable distinctions include the connection between microorganisms and the surface, large population density, and existence of an extracellular polysaccharide (EPS) slime matrix. Nevertheless, it is not arduous to locate instances of microbial communities that would be universally acknowledged as biofilms despite the absence of one or more of these characteristics. The distinguishing characteristic that distinguishes biofilm communities from planktonic cultures is their structural organization ^18^. Although the processes of biofilm development and accumulation have been established and agreed upon, researchers are now in the early stages of documenting the different types of structures and the connections between these structures and biofilm processes. An enhanced understanding of biofilm behavior is crucial because of the numerous issues linked to biofilm colonization, spanning from medical diseases to fouling of industrial components. Once formed, biofilms are highly resistant to elimination by antibiotics and other biocides. Hence, biofilm management is expensive, requires a significant amount of time, and often lacks effectiveness ^19^.

Most comprehensive examinations of biofilm structures have focused on biofilms cultivated under laminar flow conditions, even though turbulent flow is frequently more applicable to several natural and industrial processes. Owing to the fundamental impact of hydrodynamics on mass transport and fluid shear stresses, the behavior of biofilms can be changed based on the flow regime. Understanding the correlation between the form and function of biofilms as well as the elements that physically mold them is essential for optimizing the use and management of biofilms in industrial and medical environments ^20^.

## 3. Molecular Mechanism of Biofilm Formation

Membrane fouling is generally acknowledged to be primarily due to biofilm formation by microorganisms on the filter membrane surface. When several cells join, the biofilm provides a common type of growth. A biofilm is formed when well-organized bacterial cells are enclosed in an EPS matrix and attached to a solid phase. This is a complex and slow process of biofilm formation. Quorum sensing (QS) is a major component of this process ^21^. QS is a cell-to-cell signaling system in bacteria that is reliant on population density. This is recognized as a mechanism that controls bacterial communication behavior. Within a biofilm, QS can stimulate the activation of genes responsible for EPS secretion. This process also controls the physiological behavior and ecological interactions among microorganisms, ultimately influencing the form and function of the biofilm microbial community ^22^.

Despite this, some features of the regulatory mechanism of QS are still not well understood. Unlike hydrophilic signal molecules, such as acyl-homoserine-lactones, which are short chains and diffuse freely outside the cell membrane boundaries, long-chain signal molecules, however, cannot be easily removed from the cell. Furthermore, signaling molecules that are discharged into the environment may undergo degradation through the action of extracellular enzymes ^23^. Thus, to precisely transfer and convey messages between cells, it is imperative that the bacterium release a distinct vector. This vector facilitates the extracellular release of signaling molecules by cells, shields these molecules from degradation in the surrounding environment, and carries them to specific recipient cells ^24^.

## 4. Steps involved in formation of biofilms

Biofilm development is a complex procedure that involves a change from a free-swimming planktonic form into a sessile form to produce a biofilm. Temperature, pH, hydrodynamic pressures, gravitational pull, Brownian motions, quorum sensing, secondary messengers, and other signaling molecules are environmental factors that determine this process ^25^. Four main processes can be used to categorize the different stages of biofilm development.

### 4.1. Adherence

Biofilm initiation is a way of converting free-living microorganisms to cohesive communities, which begins with the surface adherence of planktonic microorganisms, and this marks it as one of the most critical stages leading to progression ^26^. In the initial phase of biofilm formation, organisms attach reversibly and loosely to surfaces. This phase is characterized by microorganisms that are in direct contact with surfaces in a polar manner. Subsequently, bacteria alter their orientation to adopt an irreversible attachment, thereby developing resistance against many physical conditions that impede the production of biofilms ^27^.

Soon after, the bacteria reorient to a flattened shape on the surfaces and commits to irreversible attachment, strengthening their resistance to various physical perturbations that present conditions not conducive for biofilm formation. Initial biofilm establishment depends on the intracellular signaling molecule bis-(3ʹ–5ʹ)-cyclic dimeric guanosine monophosphate (c-di-GMP) because it promotes biofilm matrix synthesis and suppresses flagella-driven swimming motility. The Pil-Chp surface-sensing mechanism of bacteria drives aggregated c-di-GMP concentrations during cycles of attachment and detachment ^28^. As such, the early stages of biofilm formation involve transitioning of surface-living planktonic bacteria that are naïve and have low c-di-GMP concentrations and have never encountered surfaces into surface-sensing bacteria, that is, those with high c-di-GMP concentrations that have established contact with surfaces, and cellular attachment onto a surface, which is typically irreversible, leading to biofilm formation ^29^.

### 4.2. Expansion or Creation of Microcolonies

Microorganisms adhere to surfaces and start to replicate and flocculate within the self-extracellular polymer shell shortly afterwards, which leads to microcolonies in the presence of an elevated concentration of c-di-GMP ^30^. Type IV pili and flagella-mediated motility are essential for microorganisms to engage with surfaces as well as for cell-cell aggregation to form microcolonies.

### 4.3. Maturation

EPS enhance biofilm formation because they facilitate adherence of microbes on surfaces, stabilize the three-dimensional matrix of the biofilm, aggregate cells, protect the biofilm against a number of stresses, such as those posed by the host immune system response and antimicrobials, oxidative stress, metallic cations, sequester the signaling molecules involved in quorum sensing, metabolic end-products, and enzymes necessary for this process ^31^. A mature biofilm is made up of three layers: a surface layer within which microorganisms are located and set to be shed off from the biofilm so that they can exist in a planktonic state, such as free-living bacteria, an inner layer responsible for controlling the biofilm, and a middle layer that serves as the base for microbes ^32^.

### 4.4. Spread

Finally, a mature biofilm is dispersed through two processes. The first is passive, which is driven by an external physical force such as liquid flow. The second method requires active dispersion, where motility contributes to its breakdown together with the degradation of EPS, leading to new cycle formation in biofilm production ^33^. Various factors can cause the spread of mature biofilms. These include high population, competition, sufficient nutrients, the presence of an enzyme that breaks down alginate in Pseudomonas species, EPS degradation, and cell motility promoting genes and genes reducing those for polysaccharide and fimbriae synthesis other than temperature and oxygen scarcity ^34^.

## 5. Biofilm formation on various surfaces

Biofilms, as groups or individuals, may exist in a free form of life. These consist of several species. The microorganisms in the biofilm state exhibited an ordered arrangement from the planktonic form. This is because they live together in a common EPS and may adhere to dry or wet conditions irrespective of whether the surface is living or non-living. Many differences are observed in the growth rates between biofilm-grown microorganisms and those that come alone. Over the evolution period, they have gained ways of keeping themselves away from their host by putting up some form of protective wall; they are also resistant to common antibiotics and environmental cues, such as sudden temperature changes ^35^.

The uncontrolled long-lasting nature of microbial infections is caused by persistent cells and antibiotic resistance, both of which are facilitated by the creation of biofilms. According to Datta, biofilms can be found almost everywhere and usually show several different medical signs ^36^. These are available in the human body, pipes with flowing water, pipes conveying clean water, floors within various hospital sections, places of food processing, and other abiotic and biotic surfaces. These microorganisms held by biofilms have altered phenotypic characteristics, altered gene expression patterns, less sensitivity to well-known antibiotics, and decreased rate of metabolic activity, including slow growth over time and biosynthesis of virulence factors ^37^. Biofilms are the cause–60-80% of all microbial infections.

According to NIH statistics, biofilms established on implants account for approximately 65% of microbiological tissue infections and 80% of chronic infections. These types of biofilms often infect other medical devices, such as breast implants, ventriculoperitoneal shunts, tissue fillers, left ventricular assist devices, contact lenses, catheters, joint prostheses, urinary catheters, orthopedic implants, pacemakers, mechanical heart valves, defibrillators, vascular prostheses, endotracheal tubes, and voice prostheses. Some tissue-related diseases caused by microbial biofilms include periodontitis, osteomyelitis, lung infection in cystic fibrosis, endocarditis, dental plaque, chronic tonsillitis, chronic laryngitis, chronic wounds, and biliary and urinary tract infections ^38^.

According to the 2007 statistics of the Centers for Disease Control (CDC), there were approximately 1.7 million hospital-acquired infections, over 0.5 million related fatalities, and an approximate US $ 11,000 million financial burden associated with treating biofilm-associated diseases. Furthermore, biofilm-producing microbes have a detrimental effect on a variety of food business sectors, including aquaculture, dairy, poultry, and ready-to-eat foods. This can lead to food spoilage, disease outbreaks, and fatalities ^39,40^.

## 6. EPS formation

The main constituents of EPS could be categorized as follows.

### 6.1. Polysaccharides

Although some polysaccharides undergo separation during their generation, the composition of the latter often varies. To retain the structure and stability of the biofilm matrix, polysaccharides interact with themselves as well as with proteins and ions, which involves various components such as hydrogen bonding, van der Waals interactions, electrostatic attractive/repulsive forces, and ionic attraction ^41^. Three exopolysaccharides, Pel, Psl, and alginate, are primarily responsible for *Pseudomonas aeruginosa* biofilm production and architectural maintenance. In addition to providing defense against the immune system and other external stimuli, polysaccharides function as molecular glue needed for bacterial attachment to biotic and abiotic surfaces for colonization ^42^.

### 6.2. Extracellular proteins

Extracellular proteins that are secreted combine with proteins that are subunits of cell appendages and outer membrane vesicles, and cell surface adhesins are the major components of the biofilm matrix. They are known to interact with nucleic acid components and exopolysaccharides, which enhance surface colonization, stabilize the biofilm matrix, and maintain the architecture and integrity of biofilms ^43^. Specific proteins such as proteases that degrade matrix proteins, glycosyl hydrolase dispersin B that hydrolyzes polysaccharides, and DNases that degrade extracellular nucleic acids aid in the breakdown and dispersal of the biofilm matrix. However, numerous proteins are obtained from P. aeruginosa secreted proteins and lysed cells ^44^.

### 6.3. Extracellular DNA

High concentrations of protein peptidases, disulfide isomerases, cell wall and polysaccharide metabolism enzymes, as well as chaperones (cold shock protein, DNA binding protein) have been discovered in Extracellular DNA. It has also been shown that the proteomic composition of EPS differs from that of the cell fraction. If we speak about the individual proteins in EPS matrix, it is important to mention that membrane proteins in outer membrane vesicles, also known as OMVs, make up approximately 30% of them ^45^.

In biofilms, one of the critical elements of the extracellular DNA (eDNA) matrix that enables microbial aggregation is essential. Several methods can lead to the formation of eDNA, including bacterial secretion systems, phage-induced cell death, autolysis, quorum sensing-regulated DNA release, and potential connections with DNA-containing OMVs. Human polymorphonuclear leukocytes (PMNs) produce eDNA at *P. aeruginosa *infection sites where human hosts have been infected by this bacterium, as seen in conditions such as cystic fibrosis (CF) ^46^. Chelation by eDNA results in motility control, maintenance of structural integrity, enhancement of pathogenicity by cations, and antibiotic resistance. Cell adhesion, matrix structural integrity, HGT, defense against the host immune system, and antibiotics are enhanced by eDNA through surfactants and lipids ^47^.

### 6.4. Surfactants and lipids

Certain species, such as *Rhodococcus spp.*, produce hydrophobic EPS, which clings to Teflon and colonizes waxy surfaces. revealed how biosurfactants contribute to virulence factor synthesis and heavy metal binding ^41,48^. The EPS matrix contains lipids with surface-active characteristics such as viscosin, emulsan, and surfactin. By spreading them out, hydrophobic chemicals become more available. Rhamnolipids are a significant family of surfactants that have been investigated in *Pseudomonas aeruginosa*. They assist in shaping biofilms, promoting the creation of microcolonies, and easing the dispersion of biofilms ^49^.

## 7. Regulation, Defense, and Therapeutic Challenges of Staphylococcal Biofilms

Due to its pivotal role in staphylococcal biology, biofilm formation and dissolution are tightly regulated by numerous regulatory systems that integrate the cell's physiological state and environmental signals into the dynamics of the staphylococcal community. In this context, the most investigated regulatory system is the accessory gene regulator (Agr) quorum sensing (QS) system, being a mechanism of cell-to-cell communication controlling cellular behavior based on cell density ^50^. Proteases and phenol-soluble modulins (PSMs), which are major factors in the development and disintegration of S. aureus and S. epidermidis biofilms, are primarily regulated by the QS system. With progress in biochemical techniques and new approaches for imaging, the understanding of staphylococcal biofilms has made great improvements ^51^.

Staphylococcal biofilms exhibit a great degree of complexity and spatial organization, as demonstrated by an in vitro examination of their three-dimensional structure. Furthermore, research has revealed that the composition of staphylococcal EPS varies greatly depending on the host environment, food availability, and mechanical shear pressures ^52^. While the molecular mechanisms behind staphylococcal biofilm development in vitro have been thoroughly investigated, little is known about staphylococcal biofilm formation in vivo. In vivo staphylococci are susceptible to innate host defenses, including neutrophils, macrophages, and antimicrobial peptides (AMPs), in contrast to in vitro biofilm development ^53^. Staphylococcal biofilms provide both antibiotic therapy and defence against the host immune system during infection. It is now evident that biofilms protect bacterial cells from immune system detection by hiding pathogen-associated molecular patterns (PAMPs). This contrasts with the long-held theory that biofilm recalcitrance against the immune response is caused by the biofilm microenvironment, which functions as a physical barrier for the host immune cells ^54^. Similarly, the initial theory behind biofilms was that they would stop drugs from diffusing, rendering the cells within them resistant to antibiotic therapy.

However, recent research indicates that the low metabolic activity of the cells inside biofilms may boost their resistance to antibiotics, which mainly target these metabolically active cells. Persister cells and small-colony variations (SCV) are physiologically like biofilm-associated cells with low metabolic activity ^55^. Both the Gram-negative bacteria Escherichia coli and S. aureus have been shown to have low intracellular ATP levels, which are associated with persister cell antibiotic tolerance. Low oxygen and nutrient availability cause metabolic cell activity and intracellular ATP levels to diminish in biofilm cells, which likely contributes to the biofilm's increased antibiotic resistance ^56^. Therefore, antibiofilm techniques that disrupt biofilm cells without regard to their cellular activity, such as AMPs, surface modifications that stop bacterial adhesion, antimicrobial nanoparticles, and novel technologies for physical biofilm removal, are very appealing ^57^.

## 8. Biofilm threat to public health in developing countries

Microbial biofilms were first observed by Van Leeuwenhoek, who described the presence of biofilms on the surfaces of teeth. In addition, researcher studied biofilms of microbes in industrial water systems and found that while disinfectants such as chlorine are effective in killing microbes in solution, biofilms are inherently resistant to disinfection. A biofilm generally consists of several species of microorganisms living together, and often has interstitial regions and water transport channels that penetrate the structure and allow oxygen and nutrients to enter. The growth and development of cells in biofilms are due to these factors. Recent studies have shown that resident species within biofilms obtain virulence factors that are absent in free-living bacteria ^58^. Biofilms are found in many settings, including biological tissues, medical equipment, and pipes in water systems. The microorganisms and substances formed determine biofilm establishment. Biofilms have a tendency for particle trapping of many minerals and host system components, such as RBCs, fibrin, and platelets. The growth rate in biofilms is slower than that in planktonic species. They can form aggregates of cells within the biofilm, transfer plasmid resistance between the cells, secrete endotoxins, withstand antimicrobial agents, and evade clearance by the host immune system ^59^. Biofilm adherence to structures such as pilli, flagella, glycocalyx, and fimbriae is substantially dependent on the substrate type and hydrophobicity of the cell surface. The disturbing aspect of biofilm disease in poor countries is elevated resistance to antibiotics. They are useful for the formation of slag in industrial piping, the spread of diseases in plants, and the transmission of diseases in health care environments, leading to great economic difficulties in the industry and medical fields. Many improved measures to control biofilms have been implemented. However, so far, the tactics have failed, and therefore, there is an urgent need to form new techniques ^60^.

### 8.1. Threats of Biofilm Public Health

Biofilms are ubiquitous in nature and can give rise to significant issues in both non-medical and medical domains, including the accumulation and growth of microbes (biological fouling) in portable water environment and food storage and processing settings, and medical domains, such as infections categorized into persistent and recurrent along with the ones linked with medical equipment ^61^.

### 8.2. Non-Medical Areas

Water is a vital component of human life. Universal access to sufficient and reliable water is crucial as it leads to numerous health advantages. Microbial pollution leads to numerous health issues. Developing countries are experiencing numerous significant health problems associated with the availability of safe drinking water, such as diarrhea and infant mortality, mainly in Asia and Africa. The World Health Organization (WHO) reports that the mortality rate resulting from waterborne illnesses surpasses 5 million individuals annually, with over 50% of these cases attributed to intestinal infections ^62^.

### 8.3. Biofilm formation in food industry

Bacteria, especially those that are transmitted through food, form biofilms in their natural environments, resulting in significant hygiene issues and economic losses caused by food spoiling. Microbial growth on solid surfaces is a ubiquitous phenomenon that plays a crucial role in the occurrence of food-borne illnesses and formation of biofilms in cases where appropriate sterilization is lacking ^63^. Bacterial adhesion to surfaces plays a significant role in various industries, particularly the pharmaceutical and food sectors, where L. monocytogenes is frequently encountered. Food safety is a critical public health concern that links human welfare to several aspects of food production such as farming ^64^.

### 8.4. Ready-to-eat food

Individuals in numerous nations consume ready-to-eat (RTE) and uncooked foods including marine items. E. Cloacae was the second most prevalent foodborne pathogen found in ready-to-eat (RTE) foods, according to ^65^. Similarly, the predominant pathogen found in chicken farms is S. enteritidis, which is responsible for causing foodborne illnesses in humans globally. Approximately 50% of these bacteria can produce biofilms ^66^.

### 8.5. Sea food 

Seafood-related foodborne diseases account for a considerable percentage of global hospitalizations and morbidities. This is primarily because seafood has a high nutritional content, including proteins, omega-3 fatty acids, micronutrients, minerals, and vitamins and microorganisms can easily colonize there ^67^. Seafood includes different types of marine life, such as mammals, mollusks, finfish, fish eggs, and crustaceans. Pathogens that produce biofilms mostly occur in various types of seafood, including but not limited to crabs, pacific oysters, and prawns. Seafood-borne diseases are manifestations of numerous viruses, bacteria, and parasites that develop biofilms on surfaces in contact with seafood, and water. These biofilms enable them to attach for long periods and remain resistant to many antibiotics. Exposing these biofilms to food-related stresses and environmental conditions returns them to the planktonic state ^68^. The most common microorganism responsible for contamination in fish and seafood is *Aeromonas hydrophila*, which causes resistance to antibiotics and virulence. The major contamination of seafood occurs during its handling and processing stages, which is likely to be caused by *Vibrio cholerae*. Cholera is recognized as the major cause of diarrhea in Southeast Asia, Haiti, Africa, and other poor countries. The first report of the *V. cholera O 139* epidemic was reported in 1992 in India and Bangladesh. *Salmonella spp.* are agents of infection in poultry, shellfish, dairy products, pigs, and beef. They can survive in a highly saline and high-temperature environment, making them a global threat. *L. monocytogenes* is a significant pathogen that was isolated in freshwater fish, crabs, and catfish. This virus can multiply at refrigeration temperatures after food contamination ^69^.

### 8.6. Threats of biofilms in dairy industry

The dairy sector has become one of the largest businesses worldwide owing to widespread changes in the global market ^70^. Inadequate cleaning and sanitizing in milk processing plants allows bacteria to form biofilms, which have adverse effects on both health and economic outcomes. Contrary to the sanitation and cleaning processes, it was found that bacterial cells may survive on the equipment surface. Biofilms act as a route for contamination and may cause reduced heat transfer, higher corrosion rates, and increased resistance to fluid friction. However, the quality, safety, and efficacy of dairy products are lost as soon as undesirable bacterial growth occurs ^71^. The most common bacteria in the dairy industry are typically of the genera *Enterobacter*, *Micrococcus*, *Listeria*, *Streptococcus*, *Bacillus*, and *Pseudomonas*. Milk is a good growth medium for microorganisms because of its neutral pH and nutrient-rich content. Species such as *Pseudomonas*, *Legionella*, and *Aeromonas*, which arise from rinse water, also contaminate dairy products ^72^. Biofilms in milk pipelines, milk silos, and storage tanks are another source of contamination. *Pseudomonas spp*., particularly *P. lundensis*, *P. fragi*, and *P. fluorescens*, are often culture contaminants in ultra-heat-treated (UHT) milk. These organisms produce thermolabile extracellular proteases, lipases, and lecithinases that are responsible for milk spoilage. Biofilm biofilms cause contamination of food and dairy equipment processing, lower the product shelf life, and facilitate possible cross-infections ^73^.

### 8.7. Clinical Challenges of Biofilm mediated Antibiotic Resistance Infections

The role of biofilms in the medical field is of paramount importance, as they are both a clinical challenge. Biofilms are communities of microorganisms attached to surfaces (typically medical devices, tissues, or wounds) that are encased within a self-produced extracellular matrix. Infections associated with biofilms are difficult to treat because of the high resistance of microbes to antibiotics and the host's immune system, and these structures increase the resistance of microbes. Some examples include infections associated with catheters, prosthetic joints, and dental plaques. In these contexts, biofilms are persistent, leading to chronic infections, higher healthcare costs, and surgical interventions to remove contaminated devices.

Biofilms are a nuisance in wound care as they interfere with healing and resist standard antimicrobial treatments by creating an inflammatory environment. Owing to the slow nature of wound healing, chronic wounds (e.g., diabetic foot ulcers) are particularly prone to biofilm-associated infections.

Moreover, biofilms are an increasing problem in respiratory diseases, including cystic fibrosis, as biofilms generated by *Pseudomonas aeruginosa* contribute to recurrent, severe infections ^55,74^. Bacteria in different physiological states due to nutritional gradients in biofilms contribute to the development of antimicrobial tolerance in biofilms. When nutrients and oxygen are scarce, biofilm cells modify their metabolic activities. In *P. aeruginosa*, biofilm cells exhibit heterogeneity in the physiological state of the cells they contain, unlike normal planktonic cells. Within a cluster of biofilm cells, it is possible for key nutrients and electron acceptors to be depleted in the surrounding area. Antimicrobial resistance in biofilms is influenced by the differential expression of specific genes, which is dependent on bacterial responses to local environmental conditions ^75^. Because numerous antibiotics specifically inhibit activities that take place in actively proliferating bacteria. Bacteria that form biofilms and have poor metabolic activity show heightened resistance to high concentrations of antibiotics. For example, E. coli biofilm cells may undergo physiological changes that contribute to antibiotic resistance because of the rpoS-mediated stress response. A more comprehensive understanding of the genes that exhibit differential expression during biofilm and planktonic growth conditions could facilitate the discovery of novel and efficacious therapies for illnesses associated with biofilms ^41,76^.

However, bacterial biofilms also contain persister cells that remain neither in a state of growth nor death when exposed to antimicrobial agents. Therefore, persister cells are responsible for the development of multidrug resistance. For instance, despite subjecting the *P. aeruginosa* biofilm to substantial amount of ofloxacin, persister cells remained unaffected and did not die. The persister cells exhibited greater resistance than their relatively susceptible *P. aeruginosa* biofilm counterparts. Persister cells exhibit tolerance to antibiotics through inhibition of their bactericidal binding sites and prevention of the fatal effects of antibiotics. The rationale for this phenomenon is that they generate multidrug resistant proteins that impede antibiotic targets. Persister cells are metabolically inert and exist in a dormant state. They are phenotypic variants of regular bacteria that possess a high tolerance to antibiotics without suffering any genetic changes. Persister cells arise because of several environmental stimuli including nutrition and oxygen scarcity, oxidative stress, DNA damage, and exposure to antibiotics. Persister cells maintain their viability and undergo regrowth within biofilms upon decreasing antibiotic concentration. Unlike antibiotic-resistant cells, persister cells do not grow in the presence of antibiotics. Persister cells are a unique type of cell that is different from both actively growing and stationary cells. These are the only cells that can withstand exposure to high levels of antimicrobial treatment ^77^.

## 9. Approaches to Combat Biofilm Formation

Ancient cultures have exploited the preservative and medicinal qualities of various species and herbs. By the end of the 1800s, scientists had explored the antimicrobial properties of these natural components^78^. Despite extensive research, the ability of these compounds to inhibit biofilm formation has not yet been fully verified. Recent studies have extensively examined the antibiofilm properties of several natural compounds, including plant extracts, essential oils, and honey.

### 9.1. Plant Extracts

Research has demonstrated the antibiofilm potential of several plant extracts. A Study investigated 119 plant extracts for their ability to eradicate Propionibacterium acnes biofilm and found that five extracts (*Epimedium brevicornum, Malus pumila, Polygonum cuspidatum, Rhodiola crenulata, and Dolichos lablab*) showed significant activity against it ^79^. Notably, extracts of *P. cuspidatum* and *E. brevicornum*, along with the basic components (icariin and resveratrol), exhibited sufficient biofilm-inhibiting activity, even at sub-MIC concentrations. Another study reported that *Melia dubia* bark extracts at 30 mg/mL have potential to suppress the formation of biofilm, lysis of RBC, swarming motility and hydrophobicity of *E. coli*. Similarly, a 2 mg/mL extract of *Capparis spinosa* successfully hindered the formation of biofilm and extra polymeric substances in Serratia marcescens, Pseudomonas aeruginosa, and Proteus mirabilis ^80^. Furthermore, these extracts dispersed the biofilms formed. *Lagerstroemia speciosa* fruit extracts can significantly inhibited biofilm formation by *P. aeruginosa PAO1* at 10 mg/mL^80,81^. Green tea can effectively inhibit biofilm formation by *Streptococcus mutans* and *E. coli* at varying concentrations ^82^.

### 9.2. Honey

Honey is known for its antioxidant, antibacterial, anti-inflammatory, and wound healing properties. Among a wide and diverse microbial community, honey possesses antimicrobial activity against 60 bacterial and fungal species ^83^. Recent studies have highlighted the efficacy of honey in preventing biofilm formation. Honey inhibits the formation of biofilms produced by Enterococcus spp.; thus, honey can be employed as a therapeutic agent against infections involving biofilms ^84^. Quorum sensing, virulence, and the rate of biofilm buildup by *E. coli O157* can be decreased by honey (when present in low concentrations) ^85^. Honey's antibacterial properties, combined with the presence of the antimicrobial peptide bee defensin 1, contribute to its ability to prevent biofilm formation. However, the mechanism by which honey inhibits microbial proliferation and growth remains poorly understood and necessitates further research ^86^.

### 9.3. Essential Oils

Essential oils, which are volatile substances derived from plants, have long been valued for their preservative and antimicrobial effects. Essential oils disrupt microbial cell walls, leading to the destruction of microorganisms. They are particularly promising, as they do not promote antimicrobial resistance ^87,88^. Cumin oil derived from *Cuminum cyminum* has shown efficacy against biofilm formation by *Klebsiella pneumoniae* and enhances the effectiveness of ciprofloxacin ^89^. Cinnamon oil is effective against *Streptococcus mutans*, *Lactobacillus plantarum*, and *Staphylococcus epidermidis ^90,91^*. Oregano essential oils has been shown to inhibit biofilm formation by *staphylococci *and *E. coli* and to remove active biofilms even at MIC levels ^92^. Additionally, tea tree essential oils, when combined with ciprofloxacin, significantly reduced biofilm biomass and cell numbers of *Pseudomonas aeruginosa ^93^*. Thyme oil, another essential oils, effectively inhibits biofilm development even at sublethal concentrations ^94^.

### 9.4. Bacteriophages

**Figure 1:**
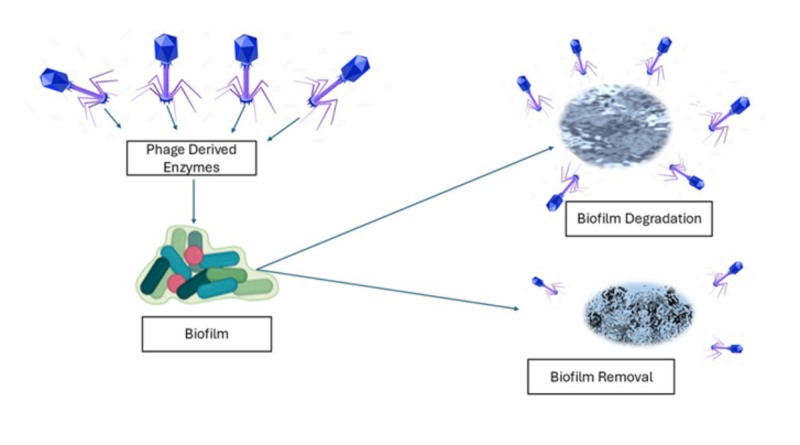
Figure 1. Schematic presentation of Bacteriophages mediated Biofilm removal

Bacteriophages, viruses that infect bacteria, have gained attention as potential alternatives or adjuncts to antibiotics, particularly for biofilm inhibition and disruption. Phages are host-specific, environmentally friendly, and can self-replicate at target sites. T4 phage, for example, effectively infects and disrupt biofilms ^95^. Phages can penetrate the EPS matrix of biofilms because they possess certain enzymes, such as polysaccharide depolymerase ^96^. Genetically engineered phages that express biofilm-degrading enzymes during infection have shown enhanced efficacy for biofilm removal ^97^. Despite their advantages, phage therapy faces challenges, such as endotoxin release and potential lysogenic conversion. However, innovative approaches address these concerns, suggesting a promising future for phage-based antibiofilm strategies (Table 1, Figure 1).

**Table 1:** Table 1. Phages and their effectiveness against Biofilm

Bacteriophage	Target Bacteria	Strain	Biofilm Type	Environment/Application	Effectiveness	Ref
T4 phage	Escherichia coli	E. coli O157	Single-species biofilm	Water treatment plants	Significant reduction in biofilm mass	^98^
Pseudomonas phage	Pseudomonas aeruginosa	PAO1	Multi-species biofilm	Medical devices (catheters)	Decreased biofilm thickness by 90%	^99^
Staphylococcus phage	Staphylococcus aureus	MRSA (Methicillin-resistant)	Single-species biofilm	Chronic wound infections	Complete biofilm eradication in treated wounds	^100^
A511 phage	Listeria monocytogenes	L. monocytogenes Scott A	Single-species biofilm	Food processing surfaces	99.9% reduction in biofilm cells	^101^
K phage	Klebsiella pneumoniae	K. pneumoniae ATCC 13883	Multi-species biofilm	Clinical settings (hospital surfaces)	Significant reduction in biofilm-forming cells	^102^
PhiIBB-PF7A	Pseudomonas fluorescens	P. fluorescens	Single-species biofilm	Industrial biofilms in pipelines	85% reduction in biofilm biomass	^103^
vB_SauM_JS25	Staphylococcus aureus	MSSA (Methicillin-susceptible)	Single-species biofilm	Dairy industry equipment	90% reduction in biofilm cells	^104^
EFDG1	Enterococcus faecalis	E. faecalis V583	Multi-species biofilm	Root canal infections	Significant reduction in biofilm viability	^105^
phiIBB-PF4	Pseudomonas fluorescens	P. fluorescens	Multi-species biofilm	Wastewater treatment	70% biofilm mass reduction	^106^
T7 phage	Escherichia coli	E. coli K12	Single-species biofilm	Laboratory biofilm models	95% reduction in biofilm cell count	^107^
PhiMR11	Methicillin-resistant Staphylococcus aureus	Staphylococcus aureus USA300	Single-species biofilm	Skin infections	80% reduction in biofilm cell count	^108^

### 9.5. Control of Biofilms with Matrix-Degrading Enzymes

Biofilm matrices, composed of DNA, proteins, and EPS, can be effectively disrupted using various enzymes. Enzymes like deoxyribonucleases, glycosidases, and proteases are crucial in breaking down mature biofilms ^109^.

#### 9.5.1. Deoxyribonuclease 1 (DNase 1)

Biofilms of gram-positive (*S. aureus and Streptococcus pyogenes*) and gram-negative (*H. influenzae, K. pneumoniae, E. coli, A. baumannii, *and *P. aeruginosa*) bacteria are affected by DNase 1 ^110^. In all organisms tested, biofilm biomass was reduced by approximately 40% after treatment with DNase 1. Additionally, when combined with antibiotics such as azithromycin, rifampin, levofloxacin, ampicillin, and cefotaxime, there was notable synergy in biofilm eradication. Additionally, DNase treatment suppressed the biofilm produced by S. aureus and P. aeruginosa ^111^, and this suppression increased up to 95% for Streptococcus pneumoniae in a dose-dependent manner ^112^. Bovine DNase 1 is effective against biofilms of *Streptococcus intermedius, S. mutans, and P. aeruginosa *^113,114^.

#### 9.5.2. Lysostaphin (LS)

Lysostaphin is a potent enzyme that invades and eradicates biofilms, particularly those formed by *Staphylococcus aureus* and *Staphylococcus epidermidis *^115,116^. Bacteria capable of generating biofilms become more susceptible to antibiotics when provided with LS in combination with oxacillin. In a murine model, LS and nafcillin, when administered together, eradicated the established *S. aureus*, including MRSA biofilms, from implanted catheters ^117^. Additionally, LS and doxycycline demonstrate significant synergistic effects against MRSA and MSSA biofilms ^118^.

#### 9.5.3. α-Amylase

Commercially available α-amylase compounds have been investigated for their ability to inhibit and remove *S. aureus* biofilm ^119^. The administration of 10, 20 and 100μg/mL μg/mL amylase decreased the rate of biofilm buildup by 72%, 89%, and 90%, respectively. Time-course experiments showed biofilm reductions of 79% and 89% within 5 min and 30 min, respectively. These findings suggest that α-amylase may be a useful tool for controlling S. aureus biofilm infections.

#### 9.5.4. Lyase

Combining lyase with antibiotics has proven effective in eradicating biofilms. For example, gentamycin (64 μg/mL) along with alginate lyase (20 μg/mL) have a potential to completely liquefy the biofilm matrix thus eradicating biofilms of two mucoid *P. aeruginosa* strains within 96 hours, reducing viable counts by 2 to 3 log10 units ^120^ .

#### 9.5.6. Lactonase

Lactonase has shown promising results in reducing biofilm formation and increasing antibiotic sensitivity in *P. aeruginosa *strains. The development of biofilm can be reduced upon utilizing lactonase (1 unit) whereas when subjected to 0.3 U/mL of lactonase, the sensitivity of P. aeruginosa to antibiotics such as ciprofloxacin and gentamycin is increased along with disruption of their biofilms. In addition, this enzyme has a capability to downregulate certain factors responsible for the virulence of p. aeruginosa including activity of protease, production of pyochelin and pyocyanin ^121^.

#### 9.5.7. Enzymes in Synergy with Surfactants and Antibiotics

Combining proteolytic enzymes with surfactants enhances biofilm wettability and cleaning efficacy. The enzymes that have an important role in this process are proteases and polysaccharide-hydrolyzing enzymes ^122^, however their widespread use is restricted because of the high cost, patent protection, and limited commercial availability of enzyme-based detergents ^123^. Nevertheless, combining different enzymes and antimicrobials/disinfectants holds promise for effective biofilm control.

#### 9.5.8. Quorum Sensing Inhibitory Compounds

Screening for quorum sensing inhibitory compounds is a promising strategy to combat biofilm-related infections. These compounds can inhibit the production or reception of autoinducers, prevent biofilm formation, or disperse established biofilms. Anti-quorum-sensing compounds are advantageous because they do not induce drug resistance and have minimal adverse effects compared to standard drugs ^124^.

*9.5.8.1. Mechanisms of Quorum Sensing Inhibition*

Enzymatic regulation of quorum sensing molecules, signal transduction shutdown, and signal receptors can be used to stop quorum sensing. For example, when halogenated furanones are emitted by the red algae *Delisea pulchra*, they are effective in inhibiting quorum sensing by interfering with the activation of the acyl-homoserine lactone-LuxR complex among gram-negative bacteria ^125^.

*9.5.8.2. Quorum Sensing Inhibitors role in controlling Biofilm*

Quorum-sensing inhibitors are important for inhibiting the formation of biofilms or dispersing biofilms. Organisms produce cyclic dipeptides as chemical signals that can stimulate or inhibit quorum sensing activities. For example, cyclo (L-Pro-L-Val) affects quorum sensing in P. aeruginosa, but the detailed mechanism is not known ^126^. In addition to bacterial species, fungi also express quorum sensing inhibitors. Farnesol from *C. albicans* inhibits the onset of germ tube and biofilm formation by inhibiting the switch of yeast to hyphal shape ^127^. In addition, nitric oxide has been considered a signal for biofilm dispersion in *P. aeruginosa* and other pathogenic microbes and is also limited by potential side effects such as immunosuppression and cytotoxicity ^128^.

Quorum-quenching compounds in combination with antibiotics improve treatment outcomes for biofilms. For example, the addition of tobramycin to patulin increases cell death in P. aeruginosa biofilms ^129^. Similarly, cis-2-decenoic acid combined with ciprofloxacin significantly improves the removal of biofilms produced by S. aureus ^117^.

## 10. Conclusion

**Figure 2:**
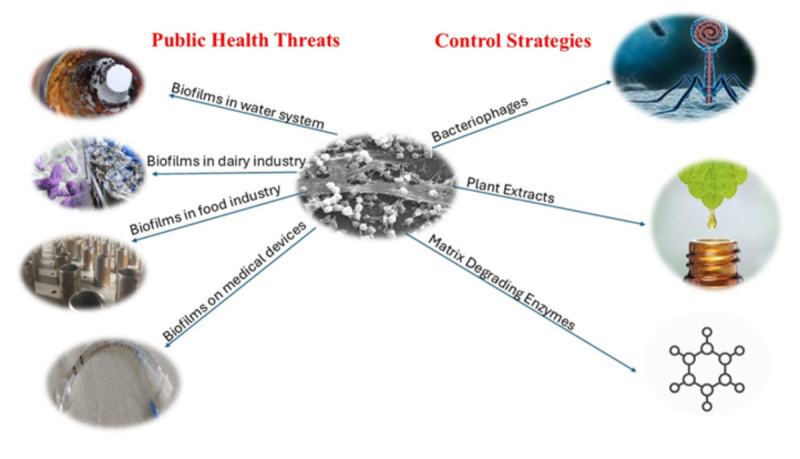
Figure 2. The multifaceted challenges posed by biofilms necessitate innovative and holistic approaches to combat their public health implications effectively

The multifaceted challenges posed by biofilms necessitate innovative and holistic approaches to combat their public health implications effectively (Figure 2). Although traditional antimicrobial treatments often fail to address biofilm-associated infections, emerging strategies offer promising avenues for intervention. Biofilm structures can be disrupted, and the efficacy of antimicrobial agents improves when specific mechanisms of their formation and persistence are targeted. In addition, the complex nature of biofilm control necessitates the adoption of comprehensive strategies, together with multiple interventions. As research continues to advance our understanding of biofilm biology and the mechanisms underlying biofilm resistance, we can further refine and optimize these strategies to mitigate the public health impacts of biofilms across diverse settings. Through collaborative efforts and continued innovation, we can address the challenges posed by biofilms and safeguard public health more effectively.
